# Reducing antibiotic use in livestock, China

**DOI:** 10.2471/BLT.19.243501

**Published:** 2020-02-28

**Authors:** Yanhong Jessika Hu, Benjamin John Cowling

**Affiliations:** a Murdoch Children’s Research Institute, Royal Children's Hospital, Department of Paediatrics, Flemington Road, Parkville Victoria 3052, Australia.; b School of Public Health, The University of Hong Kong, Hong Kong Special Administrative Region, China.

Antibiotic exposure of livestock poses risks to human health. These risks include the selection of antibiotic-resistant bacteria in livestock and their potential spread to humans by faecal-oral transmission and also through consumption of antibiotic residues in animal food products. This exposure drives antibiotic selection pressures in the human gut microbiome.

Discontinuing the use of antimicrobials for growth promotion is important for curbing antimicrobial resistance, particularly as the use of antibiotics in animals is increasing globally. With increasing antimicrobial resistance worldwide, evidence has linked human antimicrobial resistance infections to bacteria associated to livestock. For example, recent evidence indicates that the colistin-resistance gene *MCR-1* originally started in swine in China, and was later found in humans in China, and in many other countries. These findings have raised serious concerns about antimicrobial use practices in livestock. In response, many European countries have banned antibiotics for animal growth promotion.^1^ Elsewhere, such bans have been unpopular because of concerns about their impact on production capacity. However, Denmark, the Netherlands and other European countries have shown that reductions of antimicrobial use in livestock by more than half have no negative impact on production or profit. Indeed, even with lower antimicrobial use, long-term production increases are possible.^2^ A recent meta-analysis found evidence to suggest that decreasing agricultural antibiotic use could reduce antimicrobial resistance in humans by 24% (95% confidence interval: 40–60%) compared to control groups.^3^

The World Health Organization (WHO) *Global principles for the containment of antimicrobial resistance in animals intended for food,* published in 2000, stated that the use of veterinary drug feed additives should be controlled to maintain these additives’ integrity and to minimize misuse or unsafe contamination, which leads to decreased effectiveness. The Organisation for Economic Co-operation and Development, WHO, the Food and Agriculture Organization of the United Nations and the World Organization for Animal Health have been taking collective actions on policy to minimize the emergence and spread of antimicrobial resistance. In July 2019, the G20 (group of 20) ministers of health declared that urgent action was needed to tackle the global threat of antimicrobial resistance.^4^ Although antibiotic feed is banned in Europe, many low- and middle-income countries do not include these recommendations in their national agendas or are still considering them.

China is one of the world’s leading consumers of antibiotics in livestock animals.^5^ Recently, the Chinese Ministry of Agriculture and Rural Affairs launched a regulation to withdraw medicated feed additives,^6^ in accordance with the National Action Plan to Combat Antimicrobial Resistance from Animal Resources (2017–2020).^7^^,^^8^ This regulation describes three key initiatives: (i) the withdrawal of all growth-promoting feed medications except for traditional Chinese medicine; (ii) the revision of product quality standards so antimicrobials are used only for prevention or treatment, but not growth-promotion; (iii) the approval of antimicrobials only for veterinary medicine, not veterinary medicine additive purposes. The ministry aims to complete the revision of quality standards and labelling instructions by 2020.

Other countries might learn from China’s experience in formulating such a policy. However, how these plans and policies are implemented, monitored and enforced remains to be seen. For example, will reductions in antimicrobial use for growth promotion be offset by an expansion in their use for disease prevention? If implemented and enforced, would these steps be significant for containing antimicrobial resistance? Additional actions may be needed for the policy to succeed in achieving this goal, including support for those needing to convert to different business models, discouraging export of these harmful products to other countries,^9^ and public health education to encourage plant-based food consumption (or antibiotic-free animal products). This may require government investment for companies and farmers.

Without such supporting measures, implementing this policy will be challenging, as demonstrated by other South-East Asian national action plans to combat antimicrobial resistance.^10^ Additional regulations or incentives may be required to encourage alternative practices. Effective actions include vaccination and hygiene practice in animal husbandry. Such preventive interventions can lead to reduction in antimicrobial use. Some studies have shown that animals gain more weight when vaccinated.^11^ Further studies on antibiotic alternatives are needed to understand their potential for replacing antibiotics in infection control practices. Adoption of antimicrobial stewardship, essential medicine lists and antimicrobial use surveillance in veterinary practices, as in human health care, will also be critical. Further research is needed into antimicrobial replacement with non-therapeutic methods for disease prevention, including improved sanitation, hygiene, vaccination and reduction in the animal resident density. Public awareness of the dangers of antimicrobial resistance and residue exposures can increase demand for antibiotic-free products and motivate food producers to change their business practices.^2^ The WeChat social media platform, which has more than 9 million monthly active users in China,^12^ provides a useful platform to implement public education campaigns in the country. More discussions and further details on how to implement and monitor this policy and how to ensure accountability are needed. Finally, reinforcing the regulations that restrict pharmacies from selling antimicrobials over the counter, either through retail or online, is vital, as it is currently convenient for farmers to purchase antibiotics online. Although online markets are easily monitored, they can also be manipulated, and hence their regulation and surveillance pose a unique challenge. A national agriculture surveillance and monitoring system will be critical and is included in the plan, but must also contemplate investment in capacity building to ensure the action is functional and sustainable.

With China’s position as the top antibiotic producing and consuming country, actions described here are needed to help China and contribute to global antimicrobial control efforts.
